# Communicative practices and perceptions towards stuttering people in South Africa

**DOI:** 10.4102/sajcd.v71i1.1008

**Published:** 2024-03-22

**Authors:** Rockie Sibanda, Tlou C. Mothapo

**Affiliations:** 1 Department of Languages, Cultural Studies and Applied Linguistics, Faculty of Humanities, University of Johannesburg, Johannesburg, South Africa

**Keywords:** communication barriers, myths and stereotypes, people who stutter, case study, stutter experience, speech disorder, South African

## Abstract

**Background:**

A few studies have explored the life experiences of people who stutter. Research has shown that stuttering affects a significant number of people in the population.

**Objectives:**

The study was designed to explore the experiences of people who stutter and the perception of stuttering in South Africa.

**Method:**

Four people who identified as South Africans who stutter participated in this study. The primary investigator conducted semi-structured interviews with each of the participants. In addition, a questionnaire was administered to 20 acquaintances of all the participants. Transcriptions of interviews and results of questionnaires were analysed for major and minor themes.

**Results:**

Results of this study suggest different perceptions by those who stutter and those acquainted with them. The findings of the study show that people who stutter experience communication barriers, so they adopt certain strategies to manage and cope with their speech disorder. The findings showed that stuttering has a pervasive impact on the lives of people who stutter and how they view themselves, considering negative societal views.

**Conclusion:**

Evaluation of the results from the study reveals that although stuttering is a common speech disorder, many people who are less informed about it harbour various stereotypes and myths that stigmatise stuttering. This study concludes by outlining recommendations for creating awareness of stuttering. It suggests vigorous campaigns aiming at promoting a multilevel approach that extends beyond the mere social and professional understanding of stuttering but addresses the inherent perceptions, myths, and stereotypes around stuttering.

**Contribution:**

Experiences of people who stutter and perceptions towards stuttering can help to better understand the speech disorder and overcome myths and stereotyping of stuttering.

## Introduction

According to Science Daily (2018), more than 70 million people globally suffer from stuttering, and that is an estimated 1% of the world population (Cai et al., 2012). Stuttering is described as a neurobiological speech disorder (Drayna & Kang, 2011), wherein people suffer from disfluencies in speech generation. It is characterised by atypical disruptions in the flow of speech (Smith & Weber, 2017). These disruptions are attributed to genetic predisposition (Drayna & Kang, 2011), neurophysiological differences (Giraud et al., 2008; Watkins et al., 2008), differences in emotional reactivity and regulation (Jones et al., 2014; Karrass et al., 2006) or variances in speech motor control (Alm, 2004; Max et al., 2004; Namasivayam & Van Lieshout, 2008). Stuttering is increasingly recognised as a complex communication disorder and its manifestation has been gaining much traction in clinical and linguistic research globally (Brundage & Ratner, 2022; Elsherif et al., 2021; Gillam et al., 2020).

Because speech is the prime form of communication for humans, stuttering has a profound impact on multidimensional aspects of an individual’s life (Messenger et al., 2004). Speech-language pathologists who work in the amelioration of stuttering report that they do not only deal with patients presenting with impaired communication, but they are also required to understand the complex interplay of a person’s psychosocial, emotional and cultural influence on stuttering (Nang et al., 2018).

Stuttering is one of the most compelling communication disorders, specifically, in the early developmental years (Chaudhary et al., 2021), commonly affecting children from the ages of 2–6 years (Nall, 2019). Young children may stutter when their speech and language abilities are not developed enough to keep up with what they want to say (Bloodstein et al., 2021). For some children, stuttering can persist to adulthood, whereas others may recover without treatment (Bloodstein & Bernstein Ratner, 2008; Bloodstein et al., 2021; Yairi & Ambrose, 2013). Recovery is reported to be associated with the development of more complex syntactic and grammatical skills (Hollister et al., 2017; Leech et al., 2017).

However, researchers point out that evidence of recovery in childhood does not mean that there are no adults who stutter but occurrence among adults is reported to be less than 1% (Rich, 2021). When stuttering persists to adulthood, clinical intervention may be sought. If therapeutic intervention is recommended, an identifiable causal link should be established because its absence can mean that the cause of stuttering cannot be fully understood (Packman, 2012). However, Bloodstein and Bernstein Ratner (2008) report that although many treatments are available for people who stutter (PWS), they have not had considerable efficacy.

Thus, stuttering among adults is problematic when they communicate in different contexts. Complexities associated with stuttering can have a significant impact on PWS (Alqhazo et al., 2017; Bloodstein & Bernstein Ratner, 2008). They can extend beyond mere speech difficulties that individuals experience (Alqhazo et al., 2017; Bloodstein & Bernstein Ratner, 2008) to include social and psychological concerns of shame, guilt and anger, which largely stem from society’s negative portrayal and reaction to PWS (Alqhazo et al., 2017; Bloodstein & Bernstein Ratner, 2008). As already indicated, stuttering can impact PWS’s ability to interact fully in their environment, limit their ability to participate in daily activities and hurt the person’s overall quality of life (Beilbya et al., 2012; Yaruss & Quesal, 2004). Therefore, stuttering is classified as a disability.

Common misconceptions of stuttering in some parts of the world portray the condition as disabling to the affected people, thereby hindering their ability to interact and express themselves (Isaacs, 2020, 2021; Isaacs et al., 2022; Panzarino, 2019). While misconceptions underlie stuttering, various myths about the cause and cure for stuttering also exist (Denworth, 2021; Gross, 2022; Isaacs, 2021; Lei, 2022; Nicoletti, 2023). Due to the restrictive laws of apartheid, very little is known about how stuttering is perceived and managed in black communities (Mohamed & Panday, 1993 in Kathard, 1998) because little research has been documented in the area. The reported low referral rates for speech therapy in black communities (McKenzie, 1992) can, arguably, be attributed to South African history and the injustices of apartheid. Apartheid was responsible for the inequitable access to speech-language pathology services to most of the black South African population who presented with communication disorders (Moonsamy et al., 2017; Pillay et al., 1997). Consequently, various unsubstantiated beliefs surrounding stuttering demonstrate a lack of understanding of the speech disorder.

### Myths and beliefs on stuttering

Myths are designed simply for explanation, not substantive understanding. They are accounts of events that do not seek to yield knowledge but to provide comfort and aesthetic satisfaction (Wingate, 1977). Myths account for events through several assumptions, many of which have little, if any, credibility (Wingate, 1977). Some myths and beliefs are embedded in cultural and religious beliefs. Inherent cultural and religious influences cause stigmatisation and unfounded beliefs about stuttering (Haryani et al., 2020). A growing body of research on myths and beliefs on stuttering has produced several useful substantive findings. Arguably, the profession of Speech-Language Therapy (SLT) has progressed from believing that any knowledge not viewed through the lens of Western knowledge is a myth. It does not imply that if something does not comply with Western research and cannot be substantiated by ‘facts’, it is a myth. Therefore, indigenous or ‘black’ knowledge or ‘ways of being’ are not less than Western SLT professional knowledge.

Research has established a relationship between myths and beliefs around stuttering and views towards PWS. For instance, compared with other parts of the world, people in Middle Eastern countries are more likely to believe that stuttering is an act of God or a result of supernatural causes such as demons and spirits (Abdalla & St. Louis, 2012; El-Adawy et al., 2020). In India, some people believe that stuttering is the result of past life deeds (karma) of the child or their parents (Rout et al., 2014). Interestingly, Isaacs’ (2021) early enquiry about the causes of stuttering uncovered an infamous myth commonly associated with stuttering that if people mimic a person who stutters, they are likely to develop a stutter (National Stuttering Association [NSA], 2020). A related belief is that PWS played in the rain when growing up. Another myth is that if one whistled at night she or he would develop a stutter. Sande (2019) and Stanley (2019) reported that stuttering is viewed as a test of faith that needs divine intervention. In a study examining the beliefs of African indigenous healers towards stuttering, Platzky and Girson (1993) found that healers have names for stuttering, generally portraying the speech disorder as an inherited disorder. A different study on the beliefs of indigenous healers in a KwaZulu-Natal (KZN) semi-rural community identified anatomical, physiological and ancestral causes linked to stuttering (Pillay, 1992). In McKenzie’s (1992) study, interviews with community members in rural South Africa uncovered that 86% of the participants believed that stuttering was infectious and could not be cured or treated, so there was no reason to seek help. Although no formal studies investigated the complex relationship between stuttering and religion (Culatta & Goldberg, 1995), earlier anecdotal reports gathered at a KZN clinic reveal that religious explanations about stuttering exist. Therefore, the beliefs and practices of indigenous healers warrant specific consideration because as ‘psychologists, physicians, priests [*and*] tribal historians’ (Holdstock, 1979, p. 119 cited in Platszky and Girson, 1993), they are consulted by approximately 70% of the black population in South Africa (Platzky & Girson, 1993). This practice stems from several beliefs about the nature of stuttering that many people harbour. It is against this backdrop that this study is framed to investigate perceptions towards stuttering among black people in South Africa, from the perspective of those who stutter and those acquainted with them.

### Experiences of people who stutter

Several writers have profiled their experiences of stuttering and described it as disabling (Connery et al., 2020; Isaacs, 2020, 2021; Isaacs & Swartz, 2021; Isaacs et al., 2022; Panzarino, 2019). Drawing on personal experiences of stuttering, Isaacs (2021) illuminates the liminal nature of stuttering, particularly on the life experiences of a group of South African adults who stutter. A most recent study by Isaacs (2021) reflects different facets of living with stuttering and highlights the liminal nature of stuttering. As someone who stutters, the author considers the negative perception of stuttering as a moral failure, which often causes discrimination and oppression of PWS. For example, in South Africa, PWS are called derogatory names such as ‘*umangingiza*’, which denotes the regular feature of stuttering people’s verbal morphology characterised by duplication of the stem of polysyllabic words of more than two syllables. Consequently, such negative perceptions frequently make PWS feel like ‘misfits’ (Garland-Thomson, 2011). Garland-Thomson (2011) describes misfitting as an incongruent relationship between the disabled individual and the expectations of the social environment. Negative perceptions of stuttering are deeply embedded in societal views. Some PWS perceive their stuttering to have impacted their academic performance at school and relationships with teachers and classmates (Klompas & Ross, 2004; Issacs, 2021). Stuttering harmed their self-esteem and self-image. Notably, participants in the cited study indicated that although they did not consider stuttering to adversely influence their ability to establish friendships, people generally react negatively to their stuttering. Studies have shown that children who stutter tend to perform slightly below average in school (Peters & Guitar, 1991).

Research on stuttering which has been carried out in many parts of the world (Chaudhary et al., 2021) has extensively contributed to the field and increased the understanding of PWS (Plexico et al., 2009a, 2009b). A survey by Aten and Masters (2005) carried out in New South Wales, Australia, established that stuttering is a speech disorder that most people would prefer not to have. The survey discovered that many people who are ignorant about stuttering have negative perceptions towards those who stutter. As Klompas and Ross (2004) concur, the experience of stuttering may have negative effects and behavioural and cognitive reactions on one who stutters. Consequently, those who stutter can develop low self-esteem and negative self-image.

Most studies investigating the experiences of PWS have focused on men (Plexico et al., 2009a, 2009b), while a few of them have focused on women who stutter (Plexico et al., 2009a, 2009b; Beilby et al., 2013). Many studies have concluded that stuttering is more common among males than females (Bloodstein & Bernstein Ratner, 2008). Interestingly, an earlier study conducted by Craig et al. (1979) found that women who stutter exhibit higher levels of self-esteem than men, which suggests that women view stuttering as less disabling than men. Later, researchers reported that men who stutter are more likely to receive counselling or psychotherapy for their stutter because they conceive stuttering as more distressing than women (Craig et al., 2002; Silverman & Zimmer, 1982; Türkili et al., 2022). An important study, which examined how men who stutter construct their masculinities, found that men either ascribed to or rejected dominant masculine ideals or formulated affirmative masculinities in line with their speech impairment (Isaacs & Swartz, 2020). A separate study conducted by Daniels et al. (2006) found that African American men who stutter formulated certain communication identities and life choices.

Studies have associated the effects of stuttering with other facets of life such as the academic performance of affected individuals. A study by Butler (2013) found a link between stuttering and hesitancy to enrol in university. Those who attended university reported stuttering as hurting their experience of higher education and they typically avoided learning interactions requiring class discussions and seminar presentations (Butler 2013; Meredith & Packman 2015). Students who stutter have reported avoiding communicating with lecturers and peers to minimise the stress of stuttering (Meredith & Packman 2015).

In South Africa, prominent studies on stuttering are mostly school-based. For example, a study conducted by Klompas and Ross (2004) found that stuttering affects learners’ academic work and relationships with classmates. Negative perceptions and views on stuttering have been found to influence how teachers interact with learners who stutter (Abdalla & St. Louis, 2012) and influence how their peers view and treat them (Boberg & Calder, 2012; Jenkins, 2010). Thus, children who stutter are often perceived as introverts (Mallick et al. 2018). For example, a study conducted by Mallick et al. (2018) demonstrates that children who stutter are more likely to be ostracised by their peers. Other South African-based studies have uncovered varying teacher opinions and attitudes towards stuttering (Abrahams et al. 2016).

Although many PWS have profiled their personal experiences of stuttering, very little research appears to have been conducted regarding the perceived impact of this communication disorder on quality of life (Isaacs, 2021; O’Keefe, 1996). In addition, studies on communication impairments, such as stuttering, have largely been absent within disability studies (St. Pierre, 2019). Thus, the dearth of literature on stuttering and the prevailing myths surrounding it are compelling motivations for undertaking this study. As the literature suggests, many people who are less informed about stuttering harbour various stereotypes and misconceptions regarding stuttering (Isaacs, 2021). This can be attributed to the fact that the existing body of clinical literature on stuttering intervention has traditionally been derived from mainstream American populations (Shames, 1989). Therefore, this study aims to add to the existing body of literature on stuttering by exploring the experiences and perceptions towards stuttering in South Africa. To achieve this aim, the following research questions were formulated:

1. How is stuttering experienced and perceived in South Africa?
2. How does stuttering present potential barriers to communication?
3. How can awareness and understanding of stuttering be created?

## Research methods

### Participants

Purposive sampling (Creswell, 2015) and the snowball method (Creswell & Poth, 2013) were used to recruit participants. The first author used existing social networks to identify and recruit four participants who stutter. Participants were invited to participate through the personal contacts of the first author, who resided in the same community as the participants in Limpopo, South Africa. Participant eligibility criteria included a clinical diagnosis of stuttering or self-identifying as stuttering and a minimum age of 18 years. All participants stated that they started stuttering in childhood and they never had any form of speech therapy from a speech-language pathologist. The participants self-identified as stuttering for certain reasons. As research has shown, speech-language pathology across South Africa has always been inaccessible to the most vulnerable (Khoza-Shangase & Mophosho, 2021) and costly for lower-income communities to afford (Meredith et al., 2023). The acquaintances of the PWS who completed the questionnaire were either related to the interviewees or just known to them. They were recruited using the snowball method. The researcher was referred to them by the interviewees. Table 1 shows the demographic data of the four participants, and it denotes background information and speech therapy history of the interview participants. As the table shows, all the participants have a university qualification except for one with only a Grade 9 level of education. It is interesting to note that all the participants had not received speech therapy to remediate any language or speech concerns, which is further quizzed in the study. This was not an inclusion criterion but a coincidence.

**TABLE 1:** Demographic data of participants.

Participant	Age (years)	Education level	Occupation	Language	Speech and language therapy
A	26	University	IT specialist	Sepedi	No
B	24	University	Student	Sepedi	No
C	28	Grade 9	Unemployed	isiZulu	No
D	30	University	Lecturer	isiZulu	No

### Data collection and analysis

Data collection was undertaken by the first author between June and August 2021, through semi-structured interviews (Creswell & Poth, 2013; Yin, 2009). The semi-structured interview questions were generated from the research questions. They were conducted with the four participants and audio-recorded with their permission. The interview recordings were transcribed verbatim and, where necessary, translated into English by the first author. They were cross-checked for accuracy and quality by the second author. The participants were interviewed in a language and place of their choice. They preferred English, Sepedi or isiZulu, the languages with which the interviewer was acquainted. The duration of the interviews ranged from 10 min to 20 min. During and after each interview, field notes were taken, regarding the overall impression of the participants. They also included stutter behaviour patterns and the most prominent issues raised. To establish perceptions towards stuttering, additional data were collected through an online questionnaire administered to 20 acquaintances of the four participants. The self-developed 3-point Likert scale questionnaire consisted of nine closed-ended questions (Ponto, 2015). It was presented in the language of the respondents’ choice (i.e. English, Sepedi or isiZulu) and was administered online. Only three of the respondents requested a hard copy of the questionnaire, which was made available to them. Descriptive statistics were used to analyse the questionnaire results, while interview data were analysed using thematic content analysis (Maguire & Delahunt, 2017). Thematic content analysis was applied to the data because it allowed for the grouping of data into themes, which formed the main discussion for this study. During analysis, both authors checked the accuracy of the transcripts. After examining transcripts, coding was used to direct data analysis around major themes (Charmaz, 2006).

### Ethical considerations

Ethical clearance to conduct this study was obtained from the University of Johannesburg, Faculty of Humanities Research Ethics Committee (No. REC-01-092-2021). Informed consent was obtained from interview and questionnaire participants (acquaintances). All the research protocols were clearly explained to the participants, before conducting the interviews or administering the questionnaire. All participants were assigned pseudonyms to protect their identities.

## Results

This section presents data collected from interviews and the questionnaire. The first aim of the study was to explore how stuttering is experienced and perceived in South Africa. To achieve this aim, a questionnaire was administered to the acquaintances of the PWS.

The questionnaire administered to the acquaintances of PWS yielded fascinating results, showing different perceptions of stuttering. Results of the survey show low levels of understanding of stuttering (75%). A study conducted in India by Rout et al. (2014) also found that the participants in their study knew little about stuttering, but their perceptions about the cause and management were mostly fallacious. In our study, 60% of our respondents indicated that they hardly understood when speaking with someone who stutters and knew little about stuttering. Other studies have also found limited general knowledge of stuttering by many people (De Britto Pereira et al., 2008; Iimura et al., 2018; Iimura & Miyamoto, 2021; Panico et al., 2018).

Interestingly, Table 2 shows that 90% of the respondents in our study indicated that they considered stuttering to be a sensitive matter and they avoided talking about it with those who stutter. This is consistent with some African beliefs that stuttering is infectious (McKenzie, 1992; Rout et al., 2014) and discussing it can result in infection. There is somewhat of a contradiction because all the respondents indicated that they communicated with the PWS the same way that they do with those who do not stutter. However, 75% of the participants admitted to completing their sentences. In addition, 80% indicated that they considered stuttering to be a barrier to communication, which also affected how they formed and maintained relationships. Another interesting finding, emerging from the survey, is that 75% of the respondents indicated a lack of information about stuttering and 15% had moderate knowledge.

**TABLE 2:** Results of the questionnaire administered to acquaintances.

Number	Question	Response	Number of participants
1	What is your level of understanding of someone who stutters?	Hardly understand	12
		Partially understand	8
		Fully understand	-
2	How much knowledge do you think you have about stuttering?	None	17
		Moderate	3
		Much	-
3	Do you ever discuss the disorder with the person who stutters?	Yes	-
		Sometimes	2
		No	18
4	Do you consider the disorder too sensitive to discuss?	Yes	18
		A little	2
		No	-
5	Do you communicate with someone who stutters the same way as someone who does not?	Yes	20
		Sometimes	-
		No	-
6	Do you ever finish the speech of someone who stutters?	Yes	15
		At times	5
		No	-
7	Do you think stuttering can be a barrier to communication?	Yes	16
		Maybe	2
		No	2
8	Do you think there is enough awareness of the disorder?	Yes	1
		Maybe	1
		No	18
9	Do you think stuttering may affect how one relates with others?	Yes	16
		Sometimes	-
		No	4

Interestingly, 85% of the respondents indicated that when they were young, they used to laugh at their peers who stuttered, particularly when they were trying to speak in class. However, the three respondents who said that they did not laugh at PWS indicated that they either had relatives who stuttered or stuttered themselves. Interviewee C indicated that he was laughed at and teased by classmates when he was young, which led him to be reserved in class. Being reserved can be disabling to the effective use of language for learning. Notably, language is an essential skill important to children’s social and academic success (Chow & Wehby, 2019; Dickinson et al., 2010; Hulme et al., 2015). Therefore, children who begin school with language deficiency are likely to be at risk of maladaptive social behaviour and poor academic performance (Baker & Cantwell, 1987; Cohen et al., 1993; Pickles et al., 2016; Tomblin et al., 1997).

To uncover how stuttering presented potential barriers to communication, the following question was posed: *Considering your condition, is your communication different at social and professional levels?* In response, Participant A indicated that he did not see the need to change how he communicated at different levels, whereas Participant B conceded finding it ‘hard to hold conversations because of fear of being judged about how I speak’. On the contrary, Participant C reported that he found it easier to communicate at a social level, particularly with people who were familiar with his stutter. The same sentiment was shared by Participant D, who said he felt that people around him understood his speech disorder and ‘It is easier to communicate with friends socially because I can be more of myself around people I am used to’. This suggests that, because of his stutter, he was often uncomfortable and anxious around people that he was not used to, so he tried to be with those who were aware of his condition. He further reported that he stuttered most when speaking at a professional level, so he tried to speak slowly and used words that he found easy to pronounce. Research has proven that PWS are more likely to stutter less when they speak slowly (Fraser, 2010).

To establish to what extent stuttering posed a barrier to communication, the following question was posed: *How does stuttering present a potential barrier to communication*? This study found that stuttering presented a major barrier to communication. For example, interviewee B indicated that ‘people associate stuttering with being dumb and that affects my confidence’. He revealed that he had difficulties communicating in any social context because of fear of being judged about how he spoke. Subsequently, he carefully chose his words before speaking to avoid ‘swallowing words’. Further, he believed the negative views caused PWS to ‘hold back their thoughts’. Similarly, interviewee C stated that he found it ‘difficult to hold conversations with different people’, while Participant D said that he was most comfortable when speaking with familiar people because they were likely to understand his condition. He further indicated that when he stuttered, he felt less confident because he often had to repeat what he had said.

However, all the participants maintained that the barriers they experienced in communication had more to do with the attitudes and perceptions of the people they interacted with, than their speech disorder. For example, all the interviewees concurred that some of their teachers and peers were usually impatient with them and often finished their sentences, which made them feel inadequate. Participant C said that he could not pursue tertiary studies because of the low self-esteem he developed during his school days. He recounted, ‘In school, all teachers looked down on me and the kids always teased me. I just felt useless’. In addition, interviewee B indicated that his ‘confidence level got affected when stuttering’. Consequently, he barely participated in class discussions because of how people looked at him when he stuttered, which had an element of pity more than empathy. He revealed that the fear of being misunderstood grossly affected his confidence levels because, in his opinion, ‘people thought I am not smart because I stutter [*so*] I rarely spoke in class because of that’. Similarly, Participant B reported being looked down on because of his stutter. Therefore, in his opinion, people who look down on him lack empathy because ‘[*They*] look at me differently when I speak. Some even laugh when I struggle to say certain words’. Participant C shared a similar experience with Participant B, as he revealed that his stutter was most triggered by being laughed at when he spoke, and it worsened when he was upset. Similarly, Participant D also claimed that his stutter worsened with a change in his mood. Participant A concurred that he stuttered more when he was angry, so he tried to avoid being emotional. These findings suggest that different emotional states can trigger stuttering (Choi et al., 2013, 2016; Conture et al., 2013; Guitar, 2014; Yairi & Ambrose, 1992).

To uncover the barriers caused by the stuttering, the following question was posed: *Does your speech disorder present any communication barriers?* In response, Participant A claimed that he did not experience any challenges as he worked and lived with people who understood him. On the contrary, Participant B believed that people associated stuttering with being dumb, which affected his confidence. Thereby, he tended to hold back his thoughts because ‘I do not want to be looked at strangely’. Participant C said he had challenges sustaining a conversation because he was too conscious of his speech disorder, which made him anxious and stutter more. Similarly, Issacs (2021) reported that his stutter became more severe when he tried to be fluent. Studies have shown that PWS tend to stutter more when they are nervous or under pressure (Neumann et al., 2019).

Studies have shown that during adulthood, establishing intimate relationships may present unique difficulties for the adult who stutters (Ross, 2001). Although the participants were not quizzed about their romantic relationships, they shared varying experiences. Participant A revealed that a woman he dated ended their relationship because her friends teased her about dating someone who stutters, while Participant B confessed to having stopped dating because of the low self-esteem that he had developed. Participant C shared a similar experience of failed relationships. He confessed to failing to initiate and maintain romantic relationships because of being conscious of his stutter. In his opinion, the people he tried to date were judgemental and lacked understanding about stuttering in general. Participants A and D expressed similar concerns to Participant C. According to Bailey et al. (2015), a feeling of self-oppression can result in internalised oppression. Only Participant A claimed to be in a 2-month-old relationship, after a couple of failed relationships, which he attributed to negative views of his speech disorder. He reported being in a steady relationship because his partner also stutters. Having a common experience seems essential for PWS, as one participant in Klompas and Rose’s (2004) study affirms, ‘When I met a person who stutters – I felt comfortable’.

To solicit further information about the perception of stuttering, the following question was posed: *Do you think people are informed enough about stuttering? If not, what do you think should be done to get them informed?* To this question, the participants expressed different views. Participant A remarked that ‘people are only interested in what you are telling them’, whereas Participant B thought people were ill-informed about stuttering and harboured several myths such as ‘you stutter because you played in the rain growing up’. Participant C said he did not believe people are well informed about stuttering and ‘would be glad if people understand this condition and how to treat people who stutter’. In addition, Participant D indicated people make fun of those who stutter and assign them derogatory names such as ‘*umangingiza*’, which shows a lack of empathy. Participant C said some people associate stuttering with being dumb, which suggests it is an intellectual deficiency, whereas PWS are no less intelligent than people who do not stutter (Ratner & Brundage, 2021). This assertion is demonstrated in the present study because three of the four participants have tertiary qualifications.

The present study found that PWS can hold better conversations with those who are more tolerant towards their stutter than those who do not. For example, Participant A revealed that in his diverse work environment, he can easily communicate with everyone. In his opinion, he feels comfortable when he speaks because ‘people are only interested in what you are saying not how you are saying it’. This validates the understanding that PWS are not different from those who do not stutter so they are more accepting of them. The accepting listener (Starkweather & Givens-Ackerman, 1997) is likely to be patient and not ignore the ideas of those who stutter (Aten & Masters 2005). In support of this notion, Klompas and Rose’s (2004) study found that although some negative reactions were demonstrated towards participants who stuttered, they reported a positive acceptance by others.

## Discussion

The emerging themes of this study reflect the perspectives of PWS and those acquainted with them.

### Stuttering as a barrier to communication

Anxiety is cited as the main barrier to communication when PWS are speaking with people they are less acquainted with. Studies have shown that many adults who stutter have anxiety disorder and social phobia (Amick et al., 2017; Boyle, 2015; Iverach et al., 2009) when communicating with people with whom they are less familiar. Some studies have reported that families of PWS often view the liminal nature of stuttering as an invisible problem (Butler 2013; Isaacs, 2021; Scharf 2017). This suggests that the listeners’ identity can strongly affect the stuttering interlocutors’ ability to express themselves confidently. As Bloodstein and Bernstein Ratner (2008) suggest, there is a tendency for PWS to have functional difficulties of articulation. This can negatively limit their desire to express their ideas (Bricker-Katz et al., 2010).

In addition to the limiting nature of stuttering, our study found that language familiarity and proficiency have a bearing on the participants’ ability to express themselves. As Kathard (1998) notes, obtaining a thorough understanding of the participants’ idiosyncratic communicative behaviour in the speech disorder context, consideration should be made of issues of bilingualism or multilingualism. In South Africa, most of the population is bilingual or multilingual (Kashula & Anthonissen, 1995). This study established that the bi/multilingual nature of the participants influences their speech fluency. Research has found that bilingual or multilingual, the level of speech proficiency may vary between languages (Kathard, 1998). For example, all the participants in our study indicated that their speech fluency is lower when they speak English than in African languages. Kathard (1998) concurs that most Africans have reported that their proficiency in verbal communication is better in their African languages, compared with English. Sociopsychological (Nwokah, 1988), and language competency explanations (Jankelowitz & Bortz, 1996) account for such imbalances.

Related to the limiting effect of language, the participants in our study shared their unpleasant school and tertiary education experiences when they were required to express themselves verbally in class. Arguably, instruction in most South African schools is in English, which is a second language for most students, and the situation is worse for PWS, as argued previously. Isaacs (2020) affirms that negative school and university experiences can lead to hesitancy to enrol in university. A study by Butler (2013) has shown that having a stutter can have a profound effect on one’s academic achievement and progression into higher education. This view is shared by Klompass and Ross (2004) who argue that stuttering can negatively affect one’s relationships at work and home. Therefore, PWS choose to speak less. Interviewee D indicated that when he struggles to utter certain words, people look at him differently. Of the four, only Participant A claimed to be more self-confident because he believes that people are most interested in what he says and not how he says it. The fact that PWS are treated differently demonstrates a lack of information about stuttering. As Issacs (2021) observes, because PWS are treated as ‘misfits’, they are typically unable to live up to the prescripts of society and always feel shame and embarrassment.

### Challenges faced by people who stutter

Speaking a language that one is less fluent in can be challenging for PWS, and it is most likely to exacerbate stuttering. All the participants in this study revealed that they stutter more when speaking English but stutter less in their native language such as Zulu. The frequency of stuttering is imbalanced across languages. Therefore, the frequency of stuttered episodes can be greater in one language than another, depending on the level of language proficiency variances between the languages, particularly for bilingual or multilingual individuals.

Arguably, most African language speakers’ proficiency in verbal communication is better in their African languages, compared with English. Jankelowitz and Bortz (1996) have concluded that when PWS do not possess linguistic competence in a language, particularly the grammatical forms or vocabulary, they are likely to be more disfluent. For example, compared with English, Zulu is described as a tonal language, which has no monosyllabic words except for a few interjections (Kathard, 1998). It is an agglutinative language with complex and prominent morphology (Kathard, 1998). Common morphophonemic reduplication features in Zulu, which are considered a normal part of the language structure (Bailey, 1998 in Kathard, 1998), can aid in circumventing stuttering. For example, in some polysyllabic words of more than two syllables, incomplete reduplication of the stem may occur, for example, ‘They are singing’, *Bayahlabelela* and ‘They are singing a little’, *Bayahlabehlabelela*. Here, the first two syllables of the word are repeated (Bailey, 1998). Morphophonemic reduplication is not a regular feature of English verbal morphology (Kathard, 1998). Linguistic features have been an area of interest in the study of stuttering, with research findings suggesting that stuttering tends to occur in utterances that are linguistically more complex (Bloodstein & Bernstein Ratner, 2008). As Gkalitsiou and Byrd (2021) suggest, PWS do not randomly select words for production but carefully select and access words to communicate their thoughts effectively. Three of the participants in our study revealed that their inability to speak English fluently is worrying to them because English is dominant in both their social and professional interactions. In addition to language proficiency, the language preference issue has received attention in research (Kathard, 1998). In the South African context, English is perceived as an empowering language (Kashula & Anthonissen, 1995). Therefore, there is a likely preference for English within the work environment, although it is a language in which some PWS are less proficient.

### Social communicative practices

St. Pierre (2012), one of the few theorists to explore stuttering as a disability, mentions the liminal nature of stuttering when describing the disabling experiences of PWS. However, regarding the liminal nature of stuttering, St. Pierre (2012) does not view stuttering as a homogeneous phenomenon because the fluency level of PWS fluctuates across different social contexts. For example, in certain social situations, PWS may project almost fluent speech, while in other situations they may show significant levels of dysfluency (St. Pierre, 2012). The study found that PWS could communicate easily with people who were familiar with their speech disorder, which made it easier for them to express their opinions. Anecdotal evidence emanating from this study shows that PWS experience frustration when interacting at the social level. This is consistent with O’Keefe’s (1996) belief that severe communication disabilities are likely to exert a negative impact on quality of life as they cause frustration for those individuals who experience them.

### Perception of stuttering

This study uncovered negative perceptions towards stuttering. It uncovered a common belief that PWS are not smart. In the interviewees’ opinion, such beliefs demonstrate mere ignorance and stereotyping. Amick et al. (2017) observed that adults who stutter are often subject to negative perceptions that are unrelated to stuttering. The researchers found that university students perceived PWS as having lower academic competence (Amick et al. 2017). The way PWS are treated by others suggests less attention to stuttering by people in general. We found it interesting that all the respondents in our survey indicated that they perceived stuttering as a sensitive matter, like disability, and they preferred not to discuss it. St. Pierre (2012), one of the few theorists who explored stuttering as a disability, argued that stuttering is not a homogeneous phenomenon. In many African cultures, disability and illness are commonly viewed within a spiritual framework (Legg & Penn, 2013) and they are a taboo subject that is never discussed in public. Thus, the belief that stuttering is caused by an act of God is more prevalent in Africa than in North America or Europe (Abrahams et al., 2016).

### Coping with stuttering

People who stutter develop certain strategies to cope with their condition. Studies have found that PWS develop strategies to cope with stuttering or to cover up stuttering (Bloodstein & Bernstein-Ratner, 2008; Jackson et al., 2015; Guitar, 2014; Manning, 2009; Yairi & Seery, 2015). The findings of this study show that PWS adopt various coping mechanisms such as taking a deep breath before speaking, trying to speak slowly to be able to pronounce words properly and choosing words carefully before speaking. Research has found that speaking slowly is one of the most effective strategies used by PWS to cope with their speech disorder. With this style of speaking, PWS try to manage their speech disorder to accommodate other people, but they often must contend with negative social perceptions towards stuttering (Boyle, 2015). Boyle (2015) found that PWS are often classified as anxious or nervous. Such stereotyping often makes PWS avoid any form of communication (Boyle, 2015, p. 2). As Boyle (2015) argues, the stigma attached to stuttering is a result of ignorance of speech disorder.

Because of the stigma of stuttering, the interviewees in our study reported that they were often uncomfortable and anxious around unfamiliar people, so they always tried to be with those who were aware of their condition. This coping mechanism is consistent with Plexico et al.’s (2009) suggested coping strategies adopted by PWS, including avoidance, minimisation and distancing (Plexico et al., 2009). Others find ways to cover their stuttering, like remaining silent so that their stuttering cannot be observed by the listeners (Tichenor & Yaruss, 2019). It is reported that PWS can experience shame and guilt and attempt to hide their stuttering through avoidance of specific sounds, words and speaking situations (Isaacs, 2021; Murphy et al., 2007). In his famous analogy of stuttering, Joseph Sheehan (1958) suggested that stuttering is comprised of 20% overt manifestations (e.g. perceivable stuttering behaviours), and 80% covert manifestations, which include shame, guilt, fear, embarrassment, anxiety, hopelessness, isolation and denial.

The study further established that PWS devise strategies to respond to questions to avoid stuttering. The most common coping mechanism for PWS is substituting one complicated word for a simple word, pausing before trying to say a feared word, a word that can trigger their stutter (Vanryckeghem et al., 2004). Bricker-Katz et al.’s (2010) study found that PWS adopt different techniques to help them cope with their speech disorder, and overcoming their fear of speaking increased their self-confidence, leading them to communicate better. Three of the interview participants in our study indicated that they tried to breathe in before speaking and in the middle of sentences. Some research suggests that PWS have motor systems highly vulnerable to instability, which may be increased under conditions of linguistic, affective and cognitive pressure, as well as other factors, such as anxiety (Jackson et al., 2016). Other research suggests a link between the severity of stuttering and one’s body posture when speaking (Almudhi et al. 2019). It concludes that fluency of speech is likely to improve when a person who stutters’ neck and shoulder muscles are well supported.

## Conclusion and recommendations

The purpose of the study was to investigate communication practices and perceptions of stuttering. The study concluded that PWS are most misunderstood when communicating with people who do not stutter, particularly those who are unfamiliar with them. Stuttering is stigmatised due to a lack of knowledge and frivolous beliefs. To counteract negative stereotypes, PWS often adopt coping strategies to deal with stuttering. People who stutter face difficulties in their social life and education, due to intolerance by peers and educators. In addition, they often have difficulties in forming relationships because they are perceived negatively. The absence of early detection of stuttering and a lack of access to speech therapy services are common features in low-income communities.

Limitations, as well as the findings from the current study, suggest important areas for future research. Replicating this study to a larger population and a more proportionate gender representation might elicit additional data. Furthermore, the narrow scope of the current study was restrictive in exploring different perspectives of stuttering in depth. Notably, the general lack of knowledge about stuttering in most black communities of South Africa is worrying. Hence, future research needs to be undertaken in black communities to explore the perception of stuttering and the communicative practices of PWS. Respondents showed a lack of knowledge about stuttering. Vigorous awareness campaigns and documentaries of the life experiences of PWS are recommended. Filmmakers should create positive roles for PWS, instead of portraying them as clowns. Teaching about stuttering should begin at the elementary school level. In addition to creating awareness of stuttering, the government should offer a free speech therapy service to low-income communities. Participants highlighted the generally negative attitudes of people towards stuttering and the unpleasant school experiences of PWS. Hence, future research needs to also focus on the attitudes of communities, teachers, learners and family members towards stuttering and PWS. Such information could potentially enrich the field of stuttering. In conclusion, this study suggests advancing discourse in development education to help narrow the gap between PWS and community members so that the latter can have a more rounded understanding of the issues facing PWS in South Africa.
